# Biomarkers associated with anastomotic leakage after esophagectomy: a systematic review

**DOI:** 10.1007/s00423-025-03617-8

**Published:** 2025-01-28

**Authors:** Călin Popa, Diana Schlanger, Alberto Aiolfi, Moustafa ElShafei, Tania Triantafyllou, Dimitrios Theodorou, Ognjan Skrobic, Aleksandar Simic, Nadim Al Hajjar, Luigi Bonavina

**Affiliations:** 1 University of Medicine and Pharmacy Iuliu Hatieganu Cluj-Napoca, Regional Institute of Gastroenterology and Hepatology O. Fodor Cluj-Napoca, Croitorilor 19-21, 400162 Cluj-Napoca-Napoca, Romania; 2 General Surgery, Istituto Clinico Sant’Ambrogio, Milan, Italy; 3 Krakenhaus Nordwest, Allgemein-Viszeral- Und Minimal Invasive Chirurgie, Frankfurt, Germany; 4 Hippokration General Hospital University of Athens, Athina, Greece; 5 Department of Esophageal and Gastric Surgery, University Clinical Centre of Serbia, University of Belgrade, Belgrade, Serbia; 6 Division of General and Foregut Surgery, University of Milan, IRCCS Policlinico San Donato, San Donato Milanese (Milano), Italy

**Keywords:** Anastomotic leak, Biomarker, Esophageal surgery, Esophagectomy

## Abstract

**Purpose:**

Anastomotic leakage (AL) is one of the most important complications that occurs after upper gastrointestinal surgery, registering rates of 20–30% after esophagectomy. The role of systemic inflammatory biomarkers to predict anastomotic leaks is controversial and needs systematization.

**Methods:**

A systematic review based on the PRISMA guidelines criteria was performed. PubMed, Scopus, and Embase were queried using MESH Terms and All Fields key words to identify studies investigating a range of immune-inflammatory factors in predicting AL.

**Results:**

Twenty-four studies were included in this review. The total number of included patients was 5903, ranging in each study from 42 to 612. The included studies reported patients that underwent different techniques of esophagectomy (Ivor Lewis, McKeown, Orringer or thoracoabdominal esophagectomy) and 23 out of 24 studies included patients that underwent neoadjuvant treatment. While different biomarkers at different timepoints were analyzed, most studies have indicated postoperative biomarkers, between day 3 and day 5 to reach statistical significance.

**Conclusions:**

Systemic inflammatory biomarkers represent potential risk stratification and predicting tools for AL after esophageal surgery, but more studies need to be conducted to validate their clinical utility.

## Introduction

Despite the technical challenges and significant postoperative morbidity rates, esophageal resection is the cornerstone in the curative treatment of esophageal cancer [1, 2]. Anastomotic leakage (AL), one of the most feared complications of upper gastrointestinal surgery, has the potential to increase postoperative mortality and morbidity and to affect the patient’s quality of life. Anastomotic healing can be disturbed by patient and tumor-related factors, and/or by operative technique [3, 4].

The role of the immune response and systemic inflammation in esophageal cancer has been a topic of interest in medical literature in the last years. The role of different blood biomarkers, has been investigated in relation with tumor progression and cancer related survival, with promising results [5–8].

While the role of inflammation in the wound healing process is well established [9], the clinical utility of systemic inflammatory biomarkers to predict and manage AL remains quite controversial. Besides C-reactive protein and procalcitonin, other simple to use, cheaper, and largely available biomarkers and scores based on blood cell count have been investigated but there in unclear what timepoint and cutoff values are most useful. Therefore, a synthesis on the studies that discuss their role in relation with anastomotic leaks is needed.

## Materials and Methods

A systematic review of the medical literature was performed using the PRISMA (Preferred Reporting Items for a Systematic Review and Meta-analysis) criteria as guidelines [10]. The inclusion criteria for the studies were the following:

- Original prospective or retrospective cohort studies, case–control studies or randomized controlled trials
- Focus on the early diagnosis of AL using inflammatory biomarkers
- Easy to use, largely available biomarkers, based on peripheral blood cell count:
  o Leukocytes and their subtypes
  o NLR (neutrophil to lymphocyte ration)
  o LMR (lymphocyte to monocyte ratio)
  o PLR (platelet to lymphocyte ratio)
  o SII (systemic inflammatory index)
  o PNI (prognostic nutritional index)
  o CRP (C-reactive protein)
  o CRP/Alb ratio
  o Nun score (Noble and Underwood score)
  o mGPS (modified Glasgow Prognostic Score)

The exclusion criteria were the following:

- Case reports or review articles
- Articles not written in English
- Focus on other types of biomarkers
- Focus of non-serological biomarkers
- No independent analysis on esophageal cancer

The PubMed database was searched independently by two reviewers (CP and DS). Any discrepancies were resolved by consensus.

The terms of the search were ("LMR" OR "NLR" OR "PLR" OR "neutrophil to lymphocyte ratio" OR "NUN" OR "CRP" OR "C-reactive protein" OR "platelet to lymphocyte ratio" OR "lymphocyte to monocyte ratio" OR "CRP" OR "C reactive protein") AND ("anastomotic fistula" OR "anastomotic leak" OR "anastomotic leakage" OR "leak" OR "fistula" OR "leakage") ("esophagus" OR "esophageal surgery" OR "esophagectomy"). No time restrictions were used for the search. The last database search was done in January 2024.

The articles were first screened based on their title and abstract for verifying if the inclusion and exclusion criteria were met. After the first round of selection, the full-text articles were extracted and analyzed and afterwards, a decision was made whether to include the study in the review.

The title, authors, and date of publication of each included studied was noted. The data extracted from each selected study included:

- Characteristics of the study cohort: number of participants, surgical intervention details, tumor-related factors
- Morbidity and mortality reported
- Anastomotic leak rate, day of diagnosis, used definitions for anastomotic leaks
- Biomarkers analyzed in the study: timepoint for analysis, cut-off values for the biomarkers, statistical significance for AL diagnosis

The quality of the included articles was assessed using the Newcastle–Ottawa Scale (NOS).

## Results

### Search strategy

**Fig. 1:**
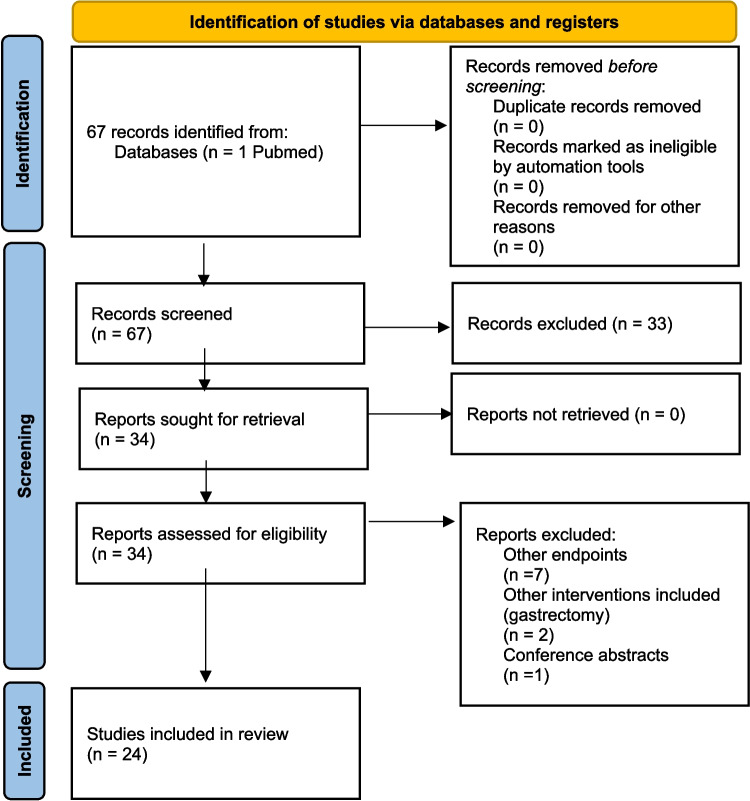
PRISMA Flow diagram – Search strategy

The search revealed 67 results; no duplicates were identified, and all records were screened. No automation tools were used for the search and screening of articles. Thirty-three more records were excluded based on their title and abstracts, before report retrieval. Thirty-four articles were assessed for eligibility and 10 articles were excluded: 7 articles addressed other endpoints, 1 article was a conference abstracts, while 2 articles analyzed other procedures, like gastrectomy. Finally, 24 articles were included in the review (Fig. 1).

The 24 included studies are presented in Table 1. The included studies were published between 2012 and 2023. Most studies had a retrospective design (21 out of 24 studies – 87.5%), and all studies were cohort studies. The total number of included patients was 5903, ranging in each study from 42 to 612. The mean age of included patients varied between 60 and 69 years. The majority of patients were male, ranging between 69 to 92% in different studies. All studies have been assessed using the NOS scale, the median score being 7.45. Eighteen studies (75%) could be classified as ‘good quality’.

**Table 1:** Characteristics of the studies included in the review

Author	Year of publication	Study design			Patients	Age	Sex (Masculine)
Hoeboer [32]	2015	Prospective	2011–2012	Netherlands	45	-	39
Park [27]	2017	Retrospective	2009–2016	Korea	201	63.9	184
Paireder [14]	2017	Retrospective	2003–2014	Austria	258	62.8	205
Asti [26]	2018	Retrospective	2012–2017	Italy	243	-	197
Noble [13]	2012	Retrospective	2005–2010	UK	258	67	202
		Prospective	2011		42	69	33
Giulini [34]	2019	Retrospective	2014–2017	Germany	80	64.4	66
Shao [25]	2019	Retrospective	2017–2018	China	450	64.26	353
Training					356	64.34	281
Testing					94	64.12	72
Al Lawati [12]	2021	Retrospective	2005–2016	Canada	330	65.6	270
Wu [11]	2021	Retrospective	2016–2020	China	198	61.3	183
Ohi [28]	2017	Retrospective	2000–2015	Japan	120	68	101
Bundred [29]	2019	Retrospective	2006–2016	UK	382	64.6	302
McAnena [24]	2019	Retrospective	2010–2016	Ireland	102	62.5	84
Liesenfeld [15]	2020	Retrospective	2010–2017	Germany	233		194
Sugimoto [33]	2021	Retrospective	2007–2020	Japan	295	65	241
Andreatta [23]	2022	Retrospective	2016–2020	Italy	121		93
Zhang [22]	2021	Retrospective	2019–2020	China	273	64.4	214
Azer [16]	2022	Retrospective	2010–2021	Germany	147	60.2	121
Hagens [31]	2023	Prospective	2016–2018	Netherlands	187	66	154
Stuart [17]	2022	Retrospective	2017–2021	Netherlands	193		134
Ri [20]	2023	Retrospective	2006–2022	Sweden	612		485
Rat [18]	2022	Retrospective	2009–2017	France	585	62.7	483
Findlay [42]	2015	Retrospective	2008–2013	UK	248	64.3	183
Edagawa [19]	2015	Retrospective	2009–2011	Japan	108	64	83
					96		79
Gao [21]	2019	Retrospective	2016–2017	China	96	62	69

### Patient and cohort characteristics

We have extracted from the included studies data regarding the tumor location, stage and histological diagnosis (Table 2) as well as data regarding the surgical intervention (Table 3).

**Table 2:** Patient and tumor characteristics

			Location					Stage—final				Diagnosis		
Study	Patients	Neoadjuvant	U	M	L	GEJ	0	1	2	3	4	SCC	AC	Other
Hoeboer [32]	45	40	N/A	N/A	N/A		N/A	N/A	N/A	N/A	N/A	11	31	3
Park [27]	201	45	31	81	89		5	85	69	42	0	201	0	0
Paireder [14]	258	161	N/A	N/A	N/A	176	N/A	N/A	N/A	N/A	N/A	82	176	0
Asti [26]	243	96	N/A	N/A	N/A		22	25	54	116	26	157	77	9
Noble [13]	258	156	1	17	96	144	N/A	N/A	N/A	N/A	N/A	N/A	N/A	N/A
	42	23	0	1	19	23	N/A	N/A	N/A	N/A	N/A	N/A	N/A	N/A
Giulini [34]	80	40	N/A	N/A	N/A		N/A	N/A	N/A	N/A	N/A	16	63	1
Shao [25]	450	375	39	271	140		0	176	96	165	13	415	6	29
Training	356	292	35	215	106		0	140	75	130	11	326	5	25
Testing	94	83	4	56	34		0	36	21	35	2	89	1	4
Al Lawati [12]	330	217	2	4	91	233	N/A	N/A	N/A	N/A	N/A	0	330	0
Wu [11]	198	0	37	96	65		0	11	69	109	6	198	0	0
Ohi [28]	120	56	11	55	54		14	31	40	24	11	108	0	12
Bundred [29]	382	294	N/A	N/A	N/A		N/A	N/A	N/A	N/A	N/A	65	297	20
McAnena [24]	102	78	N/A	N/A	N/A		7	23	37	33	2	N/A	N/A	N/A
Liesenfeld [15]	233	182	N/A	N/A	N/A	170	N/A	N/A	N/A	N/A	N/A	60	170	3
Sugimoto [33]	295	170	53	172	70		N/A	N/A	N/A	N/A	N/A	295	0	0
Andreatta [23]	121	56	N/A	N/A	N/A		8	21	23	41	20	14	86	14
Zhang [22]	273	45	20	164	89		0	104	62	101	6	256	2	15
Azer [16]	147	99	0	21	113	13	N/A	N/A	N/A	N/A	N/A	18	126	3
Hagens [31]	187	168	N/A	N/A	N/A		N/A	N/A	N/A	N/A	N/A	38	140	9
Stuart [17]	193	190	0	15	125	53	N/A	N/A	N/A	N/A	N/A	28	154	11
Ri [20]	612	437	N/A	N/A	N/A		73	69	168	167	118	124	481	7
Rat [18]	585	465	0	183	293	109	87	160	135	177	26	223	348	14
Findlay [42]	248	189	N/A	N/A	N/A		N/A	N/A	N/A	N/A	N/A	30	194	24
Edagawa [19]	108	51	9	54	41	4	N/A	N/A	N/A	N/A	N/A	98	7	3
	96	22	6	52	35	3	N/A	N/A	N/A	N/A	N/A	92	2	2
Gao [21]	96	38	N/A	N/A	N/A		0	14	46	36	0	N/A	N/A	N/A

*N/A* not accounted, *U* upper esophagus, *M* middle esophagus, *L* lower esophagus, *GEJ* gastroesophageal junction, *SCC* squamous cell carcinoma, *AC* adenocarcinoma

**Table 3:** Operative details – study cohort

		Operative time	Blood loss	Intervention						Anastomosis		Anastomosis type		Colon conduit	Jejunum conduit	Approach	
Study	No			McK	IVL	TA	Orr	EG	N/A	C	T	HS	M			Open	MI
Hoeboer [32]	45	N/A	N/A	29	0	0	16	0		45	0	24	21	0		41	4
Park [27]	201	N/A	N/A	31	170	0	0	0		31	170	31	170	0		89	112
Paireder [14]	258	330	N/A	0	199	0	34	25		34	224	N/A	N/A	N/A		167	91
Asti [26]	243	N/A	N/A	42	201	0	0	0		42	201	0	243	0		0	243
Noble [13]	258	240–242	300–350	51	112	52	43	0		94	164			N/A			
	42	N/A	N/A	1	7	N/A	6	N/A		-	-			N/A			28
Giulini [34]	80	N/A	N/A	0	80	N/A	0	0		0	80	55	25	0		16	64
Shao [25]	450	491.3	N/A	N/A	N/A	N/A	N/A	N/A		336	114	N/A	N/A	N/A		229	221
Training	356	246.4	N/A	N/A	N/A	N/A	N/A	N/A		267	89	N/A	N/A	N/A		180	176
Testing	94	244.9	N/A	N/A	N/A	N/A	N/A	N/A		69	25	N/A	N/A	N/A		49	45
Al Lawati [12]	330	N/A	N/A	66	159	73	23	0	9	89	232	313	8	4	26	266	64
Wu [11]	198	321.7	466.5	152	22	21				152	43	N/A	N/A	N/A		3	195
Ohi [28]	120	551	495	53	67	0	0	0		73	47			N/A		33	87
Bundred [29]	382	N/A	N/A	N/A	N/A	N/A	N/A	N/A		-	-			N/A		97	285
McAnena [24]	102	N/A	N/A	18	29	0	55			73	29			N/A			
Liesenfeld [15]	233	N/A	N/A	0	233	0	0	0		0	233	N/A	N/A	4	0	233	
Sugimoto [33]	295	561	300	N/A	N/A	N/A	N/A	N/A		295	0			0			
Andreatta [23]	121	360–422.5	N/A	0	121	0	0			0	121	0	121	0		1	120
Zhang [22]	273	237.6	N/A	N/A	N/A	N/A	N/A	N/A		-	-			N/A		137	136
Azer [16]	147	N/A	N/A	0	147	0	0			0	147			N/A		119	28
Hagens [31]	187	N/A	N/A	N/A	N/A	N/A	N/A	N/A		23	164	23	167	0		0	187
Stuart [17]	193	N/A	N/A		193					0	193			0		0	256
Ri [20]	612	420–431	195–200	179	374	0	59	0		-	-			0		171	441
Rat [18]	585	335.4	N/A	0	585	0	0	0		0	585	547	38	0			355 + 8
Findlay [42]	248	N/A	N/A	4	44	119	16	60		20	223			0		248	0
Edagawa [19]	108	N/A	N/A	N/A	N/A	N/A	N/A	N/A		-	-			N/A		33	75
	96	N/A	N/A	N/A	N/A	N/A	N/A	N/A		-	-			N/A		36	60
Gao [21]	96	N/A	N/A	96	0	0	0	0		96	0	9	87	0			

*N/A* not accounted, *No* number of patients, *McK* McKeown esophagectomy, *IVL* Ivor Lewis esophagectomy, *TA* thoracoabdominal esophagectomy, *Orr* Orringer esophagectomy, *EG* extended gastrectomy, *C* cervical anastomosis, *T* intrathoracic anastomosis, *HS* hand-sewn, *M* mechanical, *MI* minimally invasive

All studies except one [11] included patients which underwent neoadjuvant therapy. Eight studies [12–19] (33.33%) also included patients with tumors localized at the gastroesophageal junction. Only 11 studies [11, 18, 20–28] (45.83%) have reported the stage of the disease.

### Anastomotic leak definitions

The main outcome in all the analyzed studies was anastomotic leak. The AL definitions varied slightly between studies, however with similar meanings that referred to any disruption of the anastomosis (Table 4). Variations were mainly noted regarding the inclusion or not of the clinical significance or clinical picture in the AL definition. One article [19] did not consider the asymptomatic leaks as an endpoint, therefore excluding these patients. On the other hand, late onset AL were excluded from analysis by Azer et al. [16].

**Table 4:** Definitions of anastomotic leaks in the included studies

	AL	MORTALITY
Hoeboer [32]	Esophagoenteric leak confirmed by endoscopy or esophageal contrast videography that requires local treatment, surgical treatment, or removal of conduit	30-day
Park [27]	Anastomotic leak was defined as the disruption of the anastomosis that leads to outflow of the intraluminal content, which is obvious leaks, as well as leaks without the presence of any clinical symptoms but with only occult leaks detected with esophagography followed by chest CT	-
Paireder [14]	-	-
Asti [26]	Anastomotic leakage was suspected by the presence of clinical signs and confirmed by extravasation of oral contrast at gastrografin swallow study and/or CT scan, and/or visualization of anastomotic defect at upper gastrointestinal endoscopy	90-day
Noble [13]	AL was defined as a leak sufficient to cause symptoms and confirmed by radiology (contrastenhanced multi-detector CT scan with on-table oral contrast or water-soluble contrast studies), endoscopy or surgical exploration	in-patient
Giulini [34]	An early anastomotic leak was defined as a full-thickness lesion involving the anastomosis or the gastric conduit (staple line) requiring intervention or reiteration (grade III complication according to the Clavien–Dindo Classification14) occurring within postoperative day (POD) 5, with the rightsided chest tubes still in place	30-day
Shao [25]	AL was defined as follows: (i) the disruption of the anastomosis that leads to the outflow of the intraluminal content, which is sufficient to cause clinical symptoms and/or (ii) leaks confirmed by chest computed tomography, endoscopy, or surgical exploration	-
Al Lawati [12]	The primary outcome investigated is AL and conduit necrosis, which were diagnosed on imaging, esophagogastroduodenoscopy, or intraoperatively within 30 days of the initial operation. AL has been previously defined in the literature as a “full-thickness” GI defect involving the esophagus, anastomosis, staple line, or conduit irrespective of presentation or method of identification. Conduit necrosis was thus also included based on previous literature	-
Wu [11]	-	-
Ohi [28]	AL was defined as any esophagogastric anastomosis dehiscence that was *clinically symptomatic* (abscess, mediastinitis, externalized drainage of digestive fluid) or clinically asymptomatic but detected by contrast study within 30 days after esophagectomy, and included necrosis of the gastric conduit and anastomotic-bronchial fistulas	-
Bundred [29]	AL was classified using the same definition as the Noble and Underwood paper as any *symptomatic leak, confirmed by radiology* (computed tomography, CT, imaging or contrast swallow) or endoscopy, which required surgical, endoscopic, or radiological intervention, to be consistent with the original definition. As such, we have referred to this as ‘major AL’ throughout.* Asymptomatic or radiological leaks only requiring antibiotics or the patient remaining nil by mouth were not considered as an outcome in the primary analysis*	in-patient
McAnena [24]	AL was confirmed by radiology, endoscopy or during surgical exploration	-
Liesenfeld [15]	AL was suspected according to the presence of the following clinical signs or pathologic systemic response: fever, increased white blood cell count or CRP levels in the absence of pulmonary or urinary tract infection, development of organ failure, including respiratory or renal failure, sepsis, poor neurologic function, or gastrointestinal content within the pleural drains. In these cases, AL was confirmed according to extravasation of oral contrast at computed tomography scan and/or visualization of anastomotic defect at upper gastrointestinal endoscopy and/or surgical exploration	30-day
Sugimoto [33]	AL was defined as a full-thickness gastrointestinal defect involving the esophagus, anastomosis, staple line, or conduit, irrespective of presentation or method of identification, according to the Esophagectomy Complications Consensus Group (ECCG) consensus definition	-
Andreatta [23]	-	-
Zhang [22]	As the endpoint of this study, AL was defined as follows: (1) The leakage of intestinal content from the anastomosis led to clinical features (including intestinal contents found in surgical incision or chest tube drains, wound infection, mediastinitis, peritonitis, pneumothorax and empyema) and/or (2) Leakage was detected by imaging examination, endoscopy, or surgical exploration	-
Azer [16]	AL was defined as the passage of intraluminal content to extraluminal space through a defect in the continuity of the intestinal wall at the site of the anastomosis. Late AL (that occurred after day 14) were excluded	30-day
Hagens [31]	A type 1 leak was defined as a local defect requiring no change in therapy or treated medically or with dietary modification, a type 2 leak includes a localized defect requiring interventional but not surgical therapy, for example, interventional radiology drain, stent or bedside opening, and packing of incision, and a type 3 leak was defined as a localized defect requiring surgical therapy	in-patient
Stuart [17]	The criteria used for AL and their classification are in concordance with the recommendations of the Esophagectomy Complications Consensus Group (ECCG). AL was diagnosed after visualization of orally ingested contrast leakage on a CT scan or after a defect was visible during endoscopy or surgical re-intervention	-
Ri [20]	AL was defined as a full-thickness gastrointestinal defect involving the esophagus, anastomosis, staple line, or conduit based on the Esophagectomy Complications Consensus Group (ECCG) system	30-day/ 90-day
Rat [18]	The primary endpoint was anastomotic leak (AL), according to the International Consensus on Standardization of Data Collection for Complications Associated with Esophagectomy by the Esophagectomy Complications Consensus Group: full thickness gastrointestinal defect involving the esophagus, anastomosis, staple line of gastric conduit irrespective of presentation or method of identification, or patient’s clinical condition. AL was identified at reoperation or by the presence of air or fluid collection in the anastomotic region on CT scan or by endoscopy	90-day
Findlay [42]	AL was defined in two ways: first as Noble et al. (clinical evidence of a leak or radiological evidence plus symptoms) and second as any clinical or radiological evidence of a leak, irrespective of symptoms	in-patient
Edagawa [19]	Anastomotic leakage was diagnosed according to the definition by the Common Terminology Criteria for Adverse Events, version 4.0. Specifically, discontinuity of the esophagogastric anastomosis as detected by gastrointestinal fiberscopy, esophagography, or CT was defined as anastomotic leakage. *The clinical significance of the leakage was not considered in this study*	-
Gao [21]	Anastomotic leak was defined as a gastroesophageal defect involving esophagus, anastomosis and conduit. Therefore, gastric conduit necrosis was also classified into anastomotic leak in our study	-

*AL* anastomotic leak

The reported AL rates by each study, as well as other associated outcomes (morbidity, mortality), when reported, are presented in Table 5. The median postoperative day of diagnosis for AL was between day 4 and day 9; most reports showed the highest occurrence of AL at day 7 or day 8.

**Table 5:** Main outcomes of the included studies

Study	Patients	Complication	CD > 3	AL	% AL	AL day	Mortality
Hoeboer [32]	45	28		10	22.22%	> 4	0
Park [27]	201	N/A		23	11.44%	N/A	N/A
Paireder [14]	258	N/A		32	12.40%	9	N/A
Asti [26]	243	93	38	29	11.93%	6	11
Noble [13]	258	174	70	26	10.08%	7	7
	42	22	10	4	9.52%	6	1
Giulini [34]	80	N/A		6	7.50%	N/A	1
Shao [25]	450	N/A		55	12.22%	7	N/A
Training	356	N/A		45	12.64%	N/A	N/A
Testing	94	N/A		10	10.64%	N/A	N/A
Al Lawati [12]	330	N/A		56	16.97%	7	N/A
Wu [11]	198	N/A		25	12.63%	N/A	N/A
Ohi [28]	120	71		8	6.67%	N/A	N/A
Bundred [29]	382	N/A	123	56	14.66%	8	17
McAnena [24]	102	47		5	4.90%	7	1
Liesenfeld [15]	233	N/A		57	24.46%	7	8
Sugimoto [33]	295	N/A		34	11.53%	N/A	N/A
Andreatta [23]	121	N/A		12	9.92%	5	N/A
Zhang [22]	273	N/A		34	12.45%	N/A	N/A
Azer [16]	147	N/A		28	19.05%	7	12
Hagens [31]	187	119	36	20	10.70%	8	2
Stuart [17]	193	N/A		30	15.54%	9	N/A
Ri [20]	612	172		148	24.18%	8	17/ 30
Rat [18]	585	482	162	69	11.79%	N/A	18
Findlay [42]	248	113	46	16	6.45%	7	
Edagawa [19]	108	N/A		21	19.44%	8	
	96	N/A		23	23.96%		
Gao [21]	96	N/A		12	12.50%	N/A	

*N/A* not accounted, *CD* Clavien Dindo, *AL* anastomotic leak

### Inflammatory biomarkers

The inflammatory biomarkers had consistent definitions through studies, but different studies took into consideration different biomarkers, at variable timepoints (Table 6).

**Table 6:** List of analyzed biomarkers by each study and time points of biomarker determination in each study

Study	WCC	CRP	PCT	ALB	NUN	CRP/ALB	NLR	%N	PLR	LMR	PNI	SII	mGPS	PR	POD 0	POD 1	POD 2	POD 3	POD 4	POD 5	POD 6	POD 7	POD 8	POD 9	POD 10
Hoeboer [32]	x	x	x												x	x	x	x							
Park [27]	x	x												x	x	x	x	x	x	x	x	x	x		
Paireder [14]	x	x			x														x						
Asti [26]	x	x	x					x						x				x		x		x			
Noble [13]	x	x		x	x											x	x	x	x	x	x	x			
Giulini [34]		x														x	x	x							
Shao [25]	x	x		x		x								x				x							
Al Lawati [12]							x							x	x	x	x	x							
Wu [11]				x			x		x			x		x		x	x	x	x	x	x	x			
Ohi [28]		x		x			x							x											
Bundred [29]	x	x		x	x											x	x	x	x	x	x	x			
McAnena [24]		x												x		x	x	x	x	x	x	x	x	x	x
Liesenfeld [15]	x	x			x										x	x	x	x	x	x	x	x			
Sugimoto [33]		x		x		x					x		x	x											
Andreatta [23]		x													x			x		x		x			
Zhang [22]		x		x		x	x		x	x	x							x							
Azer [16]	x	x														x		x		x			x		
Hagens [31]		x																x		x		x			
Stuart [17]		x														x	x	x	x	x					
Ri [20]		x															x	x	x						
Rat [18]		x																x	x	x					
Findlay [42]	x	x			x														x						
Edagawa [19]	x	x														x	x	x	x	x	x				
Gao [21]	x			x												x	x	x	x	x	x				

*WCC* white cell count, *CRP* C-reactive protein, *PCT* procalcitonin, *ALB* albumin, *NUN* Noble and Underwood score, *NLR* neutrophil to lymphocyte ratio, *N%* neutrophil percentage, *LMR* lymphocyte to monocyte ratio, *PLR* platelet to lymphocyte ratio, *SII* systemic inflammatory index, *PNI* prognostic nutritional index, *SII* systemic inflammation index, *mGPS* modified Glasgow prognostic score, *PR* preoperative, *POD* postoperative day

Table 7 shows every biomarker reported to have a significant power to predict anastomotic fistula. While a few studies indicated preoperative biomarkers, most studies have indicated postoperative biomarkers, between day 3 and day 5 to reach statistical significance.

**Table 7:** Biomarkers with statistical significance

STUDY	MARKER	DAY
Hoeboer	WCC	-
	CRP	3
	PCT	1
		3
	CRP	0–3
Park	CRP	3
Paireder	Nun	4
Asti	CRP	3
		5
	PCT	3
		5
	WCC	3
		5
	%N	3
		5
Noble		
Development data set		
	WCC	4–7
	CRP	2–7
	Alb	3–7
	Nun	3–7
Validation data set	WCC	5
	Nun	2–5
Giulini		N/A
Shao	Ly	3
	CRP	3
	Alb	3
	CRP/Alb	3
Al Lawati	NLR	3
	NLR trend	
Wu	N	Preop
	Ly	1–3
	NLR	Preop + 1–3 + 4–7
	PLR	1–3
	SII	Preop + 1–3
Ohi	NLR	Preop
Bundred	CRP	4
	Alb	4
	Nun	2–7
McAnena	CRP	Preop + 3–4
Liesenfeld	WCC	3–5
	CRP	3–7
	CRP trend	1–3, 2–3, 2–4, 3–4, 2–5, 3–5
Sugimoto	CRP	Preop
	CRP/Alb	Preop
Andreatta	CRP	3, 5, 7
Zhang	CRP/Alb	3
	PLR	3
	PNI	3
	Alb	3
Azer	WCC	3, 5, 8
	CRP	5, 8
Hagens	CRP	3, 5, 7
Stuart	CRP	3–5
Ri	CRP	2–4
	CRP trend	2–3, 3–4
Rat	CRP	3–5
Findlay	WCC	4
Edagawa	CRP	3–6
	FIB	3–6
Gao	preAlb	4–5

*WCC* white cell count, *CRP* C reactive protein, *PCT* procalcitonin, *Nun* Noble and Underwood score, *%N* neutrophil percentage, *N* neutrophils, *Ly* lymphocytes, *Alb* albumin, *NLR* neutrophil to lymphocyte ratio, *PLR* platelet to lymphocyte ratio, *SII* systemic inflammatory index, *PNI* prognostic nutritional index, *FIB* fibrinogen, *preAlb* prealbumin

The cut-off values for the different biomarkers used in the included studies are reported in Table 8. The most commonly reported biomarkers were the following:

- White blood cell count
  - Postoperative day 3 – cutoff between 11–12.4
  - Postoperative day 4 – cutoff between 6.8–15
  - Postoperative day 5 – cutoff between 8–8.9
- CRP
  - Postoperative day 3 – cutoff between 59–229 mg/dl
  - Postoperative day 4 – cutoff between 110–190 mg/dl
  - Postoperative day 5 – cutoff between 83–188 mg/dl
- CRP/Alb
  - Postoperative day 3 – cutoff between 3–4.25
- NLR
  - Postoperative—Cutoff between 9.5–10
- Nun score
  - Postoperative day 4–5 – cutoff 10

**Table 8:** Reported cut-off values

	MARKER	DAY	CUTOFF	P	SN	SP	PPV	NPV	AUC
Hoeboer	WCC	-	-						
	CRP	3	229	0.002	71	84	50	93	
	PCT	1	1.82	0.005	22	100	100	83	
		3	0.35	< 0.001	67	80	55	87	
	CRP trend	0–3	55	< 0.001	80	80	50	94	
Park	CRP	3	171.2	< 0.001	69.2	78.1			0.822
		nonNt + Nt	164.2	0.042	80	70			0.71
Paireder	Nun	4	10		45.2	73.8	19.4	90.6	
			7.6		93.6	5	12.1	84.6	
Asti	CRP	3	181		63	74.1			0.732
		5	83		89.3	60.8			0.818
	PCT	3	0.672		70.6	61.7			0.672
		5	0.38		77.8	71.4			0.751
	WCC	3	12,420		34.5	91.3			0.625
		5	8950		58.6	83.7			0.694
	%N	3	78.9		76.9	56.6			0.683
		5	73.4		68.4	70.7			0.692
Noble									
Development data set	WCC	5	8.95		78	58			0.715
	CRP	5	188.5		78	63			0.751
	CRP	4	180		75	47			0.685
	Alb	5	22.5		76	56			0.742
	Nun	5	10		88	55			0.796
	Nun	4	10		95	49			0.801
Validation data set	Nun	4	10		100	57			0.879
Giulini	CRP	1–3	300		75	85			
Shao	CRP/Alb	3	4.25						0
Al Lawati	NLR	3	10		74.5	58	25	92.4	0.704
	NLR trend	3–1							
Wu	N	Preop	4.1	0	60	66.5			0.737
	PLR	1–3	220.1	0.021	88	39.3			0.643
	NLR	4–7	9.5	0.038	72				0.628
Ohi	NLR	Preop	2.5	0.05					
Bundred	Nun	4	10	< 0.001	73	65		95	0.77
McAnena	CRP	3	201.5	0.02					0.781
	CRP	4	125.5	0.009					0.755
Liesenfeld	WCC	3	11	0.005	44	76	35.1	82.1	0.65
		4	8	0.0005	64	58	31.3	84.6	0.67
		5	8	0.021	65	53	28.9	83.5	0.55
	CRP	3	150	0.014	72	44	27.6	84.3	0.6
		4	145	0.0001	68	63	35.1	86.9	0.65
		5	155	0.0001	46	71	31.7	81.6	0.6
		6	120	0.0004	64	50	27.4	82.4	0.53
		7	140	0.002	57	51	25.5	80	0.52
	Nun	4	10				31.3	78.7	0.68
Sugimoto	CRP	Preop	0.6	0.02	85.2	34.5			0.598
	CRP/Alb	Preop	1.39	0.007	88.2	34.9			0.617
Andreatta	CRP	3	225	0.007	56	92	36	93	0.772
		5	157	< 0.0001	83	87	40	98	0.848
		7	154	< 0.0001	80	96	62	96	0.937
Zhang	CRP/Alb	3	3	< 0.001	88.24	90.38			0.943
	PNI	3	58.9	0.034	52.94	69.38			0.612
Azer	WCC	3		< 0.01					0.669
		5		< 0.01					0.665
		8		< 0.01					0.682
	CRP	5		< 0.01					0.672
Hagens	CRP	3	141	0.001	79	61	20	96	0.733
		5	120	< 0.001	75	82	32	97	0.825
		7	137	< 0.001	71	83	34	96	0.789
Stuart	CRP	3	59	< 0.001	93				0.733
		4	110	< 0.001	90				0.762
		5	106	< 0.001	90				0.789
Ri	CRP	2	211	< 0.001	38.5	77.9	35.9	79.8	0.607
		3	222	< 0.001	47.3	76	38.9	81.7	0.655
		4	190	< 0.001	56.2	73.2	40.4	83.8	0.668
		2–3	4.65%	< 0.001	64.9	71.3	56.1	78.3	0.616
		3–4	25.70%	0.026	88.6	21.1	41.9	74.2	0.636
Rat	CRP	3	170		85	53		96	0.68
		4	155		79	54		95	0.69
		5	130		87	51	21	96	0.7
Findlay	WCC	4	6.89		93.8	20.7			0.641
			15	0.021	6.3	97			0.641
Edagawa	CRP	3	86.2	< 0.01	71.4	58			
	FIB	4	712 mg/dl	< 0.01	52.4	89.5			
Edagawa Validation	CRP	3	86.2	0.019	75	42			
	FIB	4	712 mg/dl	0.527	16.7	91.5			
Gao	preAlb	5	128	0.046	100	50			0.825

*SN* sensitivity, *SP* specificity, *PPV* positive predictive value, *NPV* negative predictive value, *AUC* area under the curve, *WCC* white cell count, *CRP* C reactive protein, *PCT* procalcitonin, *Nun* Noble and Underwood score, *%N* neutrophil percentage, *Alb* albumin, *NLR* neutrophil to lymphocyte ratio, *PLR* platelet to lymphocyte ratio, *PNI* prognostic nutritional index, *FIB* fibrinogen, *preAlb* prealbumin

### Intrathoracic versus cervical anastomosis

Most articles included in this review (62.5%—15 out of 24) included patients operated through different surgical approaches, without presenting a subanalysis based on the localization of the anastomosis. Three articles [19, 29, 30] did not mention at all the surgical technique (Table 3). Eight articles [11, 12, 14, 20, 25–28] showed there is no statistical difference in the AL rate between patients with different types of esophagectomies or based on the localization of the anastomosis. One article by Hagens et al. [31] reports a significantly higher AL rate in cervical anastomosis compared to intrathoracic anastomosis.

Eight articles restricted the inclusion of patients based on the used surgical technique – 3 articles [21, 32, 33] included only patients with cervical anastomosis, while 6 articles [15–18, 23, 34] only included patients with intrathoracic anastomosis. Based on this subgroup of patients, we report in Table 9 the reported AL and cutoff values of postoperative day 3 CRP level in patients with cervical anastomosis versus intrathoracic anastomosis.

**Table 9:** Cervical versus intrathoracic anastomosis

Intrathoracic anastomosis			Cervical anastomosis		
Study	% AL	CRP POD 3	Study	% AL	CRP POD 3
Giulini [34]	7.50%	300	Hoeboer [32]	22.22%	229
Liesenfeld [15]	24.46%	150	Sugimoto [33]	11.53%	225
Andreatta [23]	9.92%	225	Gao [21]	12.50%	-
Azer [16]	19.05%	-			
Stuart [17]	15.54%	59			
Rat [18]	11.79%	170			

*AL* anastomotic leak, *CRP* C reactive protein, *POD* postoperative day

## Discussion

Anastomotic leakage represents a serious complication of upper gastrointestinal surgery which significantly affects the postoperative course of patients. Anastomotic leaks occur in up to 20–30% of patients after esophagectomy [2, 35, 36]. Early recognition of AL is crucial in order to reduce its associated morbidity and mortality and may favorably impact the postoperative course by excluding patients from fast-track pathways, selecting patients for further diagnostic studies, and tailoring the therapeutic interventions [2].

Several inflammatory biomarkers measured from blood and peritoneal/pleural fluid samples have been studied in an effort to improve the diagnosis of AL, including C-reactive protein, procalcitonin, amylase, etc. However, the results of these markers showed a variable predictive value [37].

The role of inflammatory biomarkers as predictors for survival has already been studied, without clear predictive power or clinical applicability [38, 39]. However, they might still be useful as prognostic factors in other aspects of the postoperative evolution of patients undergoing esophageal resections. The purpose of this review was to analyze the potential role of systemic inflammatory biomarkers as a diagnostic tool for predicting AL following esophageal surgery, used to complete the initial screening of patients who will benefit from early imaging, and early treatment. The need for effective markers for AL is undeniable, but requirements like being easy-to-use and widely available are mandatory for wide implementation in clinical practice.

Our extensive literature search has shown few results, underlining the fact that this is a fairly new subject, mostly being explored in the last 10 years since the included studies were published 2012 and 2023. Most studies (87.5%) were retrospectively designed which brings along some limitations in data interpretation. A high variability in study design can be observed from the diagnosis of the included patients to the reported outcomes. While the literature research was restricted to esophageal cancer patients, the tumoral and operative characteristics vary between centers; this heterogeneity actually reflects the reality of esophageal cancer patients and the variability among them.

The patients’ characteristics, regarding the tumor as well as the surgical intervention were collected and structured into tables. Most studies reported part of these details, but we can see that different tumor stages and histology, as well as different surgical interventions (including the localization of the anastomosis – cervical or intrathoracic) were analyzed together. Unfortunately, there is no way to interpret the role of the biomarkers on patient subgroups, so the analyzed biomarkers need to be seen as available for the entire cohort of esophageal cancer patients undergoing resection procedures, irrespective of their particularities. This may be considered as an advantage since it offers an overview on the heterogenous cohort of patients that esophageal cancer reflects.

An in-depth discussion based on the localization of the anastomosis after different surgical techniques (cervical or intrathoracic) would be helpful to better understand the dynamic of inflammatory biomarkers on these subgroups; however, most studies do not independently analyze these subgroups. On the other hand, the reported AL rate between different surgical techniques for esophagectomy are similar in most studies. Therefore, the dynamic of the studied biomarkers should be seen as applicable on the heterogenous cohort of patients undergoing esophagectomies. Furthermore, the systemic inflammatory response occurs after AL irrespective of its localization – these biomarkers would be useful as diagnostic tools to identify high-risk patients for developing AL.

We have decided to evaluate all known inflammatory biomarkers at different timepoints, as reported by different studies: this analysis, although not unform and difficult to compress in one study is very useful to determine the clinical relevance and applicability of these data.

Since our review analyses predictive biomarkers for AL after esophageal surgery, the reported postoperative outcomes of each study need to be discussed as well. We have gathered the reported AL rates between studies, ranging from 4.9% to 24.4%, rates which align with the already known reported data in medical literature. Other postoperative morbidity and mortality data were not constantly available, since the main focus of these studies was the prediction of anastomotic leak.

White blood cell count, C-reactive protein and procalcitonin are widely used inflammatory markers that are used in daily practice, but their specificity towards different postoperative complications is unknown. However, some studies included in this review linked high WCC, high CRP and high PCT with increased risk of developing anastomotic leakage. The fluctuation of these values has also been a matter of discussion in an effort to enlighten the trend towards lower or higher numbers throughout the postoperative course of these patients and predict the response to treatment.

NLR is one of the most well-known inflammatory biomarkers and one of the first ones used. The dynamic of the neutrophil and lymphocyte fractions have been proven to play roles in carcinogenesis, host immune response and cancer progression [30, 40, 41], making NLR a promising oncologic biomarker; on the other hand, the few studies that are gathered in this review are the only ones that assess the role of the same biomarker in the prediction of postoperative complications after surgery for esophageal cancers.

Other scores based on peripheral blood counts, albumin, and CRP, like PLR, PNI and CRP to albumin ratio although less discussed, showed promising results and invite to further investigation. The Nun score showed good performance parameters and had also the advantage of being tested at fix timepoints in different studies (postoperative day 4), and fix cut-off value (10) assuring uniform results between studies.

Regarding the performance of different biomarkers, most of them showed a high negative predictive value, which offers the possibility to identify patients at low risk for anastomotic leak and therefore, apply enhanced recovery protocols selectively to these patients.

Different time points for blood sample collection have been seen, with different goals for biomarkers calculated based on these time points. Preoperative biomarkers are useful in stratifying high or low risk patients for AL, highlighting the patient subgroups that need preoperative optimization. On the other hand, a biomarker based on postoperative data is useful in the early diagnosis of AL, to identify patients for which supplementary imaging investigations are advised or when targeted treatment should be initiated. Although inflammatory biomarkers were determined based on different time points in relation to the surgical intervention and serve different purposes, each of them can find a significant place in the clinical management of patients undergoing upper gastrointestinal surgery.

The similar values of biomarkers between studies show that finding a universal cut-off value applicable in clinical practice is not impossible and should be the main goal of future studies. Cut-off values should be studied in specific time intervals in preoperative settings as well as postoperative setting, in order to solve both the issue of risk stratification as well as the early prognosis. Certain time frames and cut-off values for them should be further investigated based on the promising results showed by the included studies in this review. For example, the Nun score, on day 4, with a cut-off value of 10 indicated good predictive value and needs further validation. WCC in postoperative days 4–5 with a cut-off value of around 8 × 10^9^/l can be of clinical significance. Postoperative CRP values showed more variation regarding the ideal cut-off, but some patterns can be seen: cut-off values for CRP on day 3 of around 150–230 mg/dl and on day 5 of around 100–160 mg/dl should be further investigated. Postoperative NLR values were discussed only in two studies, but with similar cut-off values 9.5–10.

An analysis based on the CRP levels on postoperative day 3 in studies that only analyzed a specific subgroup of patients based on anastomosis localization, allows a short comparative discussion on biomarkers in cervical versus intrathoracic anastomosis. The reported values are diverse, but it can be observed that most studies report similar values between 150 and 300 as cutoff points. Even though clinically, the impact of intrathoracic AL should be higher, this is not evident in the systemic inflammatory response.

All the discussed biomarkers are based on routine blood testing after esophageal surgery, but even though widely used, there are not yet exact values available to indicate the possibility of occurrence of a certain complication or to guide the medical decisions based on the results of the bloodwork. Therefore, insisting on this subject is of utmost importance. Even though one biomarker is not perfect, seeing the broad range of possibilities might help create complex scoring systems using more biomarkers together, increasing their sensibility and specificity. Specifically, artificial intelligence algorithms might help integrate all this data, compile them together, easy the interpretation and offer support in the clinical decision-making process.

There are several limitations in our study. The major bias is the retrospective design of most included studies. Other limitations are the heterogeneity about patients’ characteristics, treatment modalities, timing of blood sampling and cut-off thresholds.

Based on the findings of the current review, future prospective studies should identify universal cut-off values of inflammatory biomarkers in order to increase the potential of clinical application. The systemic inflammatory biomarkers should be analyzed comparatively together with other known biomarkers and predictive factors for AL. It would be also interesting to address the utility these biomarkers in predicting AL after gastro-esophageal surgery performed for benign conditions, including bariatric surgery.

## Conclusions

Systemic inflammatory biomarkers may provide effective risk stratification and have the potential to influence postoperative management. Several biomarkers are available and a clearer image on their clinical applications is starting to contour. Different biomarkers can help stratify esophageal cancer patients that undergo esophageal resections depending on their risk to develop an anastomotic fistula. Further investigations are needed to establish the exact cut-off values and timepoints for these biomarkers.

## Data Availability

No datasets were generated or analysed during the current study.
